# Describing heart rate variability in patients with chronic atrial fibrillation during hospitalization for COVID‐19

**DOI:** 10.1002/joa3.12569

**Published:** 2021-06-17

**Authors:** Joey Junarta, Joshua M. Riley, Behzad B. Pavri

**Affiliations:** ^1^ Department of Medicine Thomas Jefferson University Hospital Philadelphia PA USA; ^2^ Sidney Kimmel Medical College Thomas Jefferson University Philadelphia PA USA

**Keywords:** atrial arrhythmias, atrial fibrillation, COVID‐19, electrophysiology, heart rate variability

## Abstract

**Introduction:**

Myriad cardiovascular manifestations have been reported with COVID‐19. We previously reported that failure of PR interval shortening with increasing heart rate (HR) in patients with COVID‐19 is associated with adverse outcomes. Here, we report on heart rate variability (HRV) and clinical outcomes in patients with chronic atrial fibrillation (cAF) hospitalized for COVID‐19.

**Methods:**

A retrospective review of admitted COVID‐19 patients with cAF between 1 March 2020 to 30 June 2020 was performed. HRV in cAF was compared during pre‐COVID‐19 and COVID‐19 admissions; we selected pre‐COVID‐19 ECGs with HRs that were within 10 beats per minute of the COVID‐19 ECGs. Mean HR and each RR interval were recorded. Time‐domain measurements of HR variability were then calculated (SDSD, RMSSD, pNN50). Clinical outcomes during COVID‐19 were correlated to indices of HRV.

**Results:**

A total of 184 ECGs (95 pre‐COVID‐19, 89 COVID‐19) from 38 cAF in‐patients were included. Mean age 78.6 ± 11.4 years, male 44.7%. The mean number of ECGs analyzed per patient pre‐COVID‐19 was 2.50 and during COVID‐19 was 2.34. Comparing pre‐COVID‐19 versus COVID‐19 ECGs showed: mean HR (95.9 ± 24.3 vs. 101.6 ± 22.8 BPM; *P* = .10), SDSD (109.0 ± 50.6 vs. 90.3 ± 37.2 ms; *P* < .01), RMSSD (184.1 ± 80.4 vs. 147.3 ± 59.8 ms; *P* < .01), pNN50 (73.8 ± 16.3 vs. 65.6 ± 16.6%; *P* < .01). Patients who had a smaller pNN50 during a COVID‐19 admission had increased mortality (50.0% vs. 14.3%; log‐rank test *P* = .02).

**Conclusion:**

In patients with cAF, the HRV was reduced during COVID‐19 compared with prior illnesses at similar average heart rates. Patients with the most depressed HRV as measured by pNN50 had an associated increase in mortality compared with patients whose HRV was preserved.

## Graphical abstract

The electrophysiologic effects of COVID‐19 in patients with chronic atrial fibrillation (cAF) have not been described. Anecdotally, we observed that patients hospitalized for COVID‐19 with comorbid cAF demonstrated more regularized ventricular rates. This observation prompted further analysis of heart rate variability (HRV) and clinical outcomes in patients with cAF hospitalized for COVID‐19. We found that in patients with cAF, the HRV was reduced during COVID‐19 compared with prior illnesses at similar average heart rates. Patients with the most depressed HRV as measured by pNN50 had an associated increase in mortality compared to patients whose HRV was preserved. Thus, the depressed HRV in AF may reflect changes in autonomic control of AV conduction, and if confirmed, may be another marker of cardiac injury in COVID‐19.

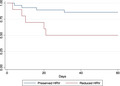

## 1 INTRODUCTION

The coronavirus disease 2019 (COVID‐19) pandemic has resulted in considerable morbidity and mortality. Although the disease predominantly affects the respiratory system, multiorgan dysfunction, including cardiac injury, is widely reported. Cardiac injury is common in COVID‐19 patients, with studies reporting a prevalence of 19.7%, 29%, 77%, in hospitalized, critically ill, and deceased patients, respectively.^1^, ^2^, ^3^ Various disease manifestations, including myocarditis, acute coronary syndrome, arrhythmias, heart failure, and venous thromboembolism have all been reported.^4^ Importantly, cardiac involvement is associated with a higher risk for in‐hospital mortality, and patients with pre‐existing cardiovascular abnormalities have an increased risk for severe illness.^1^, ^5^ It has been proposed that the virus causes direct myocardial injury, whereas the release of cardiotoxic cytokines further exacerbates this injury.^6^

Currently, there is still a paucity of literature detailing the effect of COVID‐19 on the cardiac conduction system and autonomic nervous system (ANS). We have previously reported that in sinus rhythm, the PR interval in patients with COVID‐19 may fail to shorten appropriately with increasing rate and that this observation is associated with adverse clinical outcomes.^7^ Electrophysiologic effects of COVID‐19 in patients with chronic atrial fibrillation (cAF) have not been described.

Anecdotally, we observed that patients hospitalized for COVID‐19 with comorbid cAF demonstrated more regularized ventricular rates. This observation prompted further analysis of heart rate variability (HRV) and clinical outcomes in patients with cAF hospitalized for COVID‐19.

## 2 METHODS

### 2.1 Study population and design

We performed a retrospective review of all admitted COVID‐19 patients with the diagnosis of cAF between 1 March 2020 and 30 June 2020 at Thomas Jefferson University Hospital in Philadelphia, USA. Clinical and electrocardiographic (ECG) data were collected from a shared electronic health records system and the hospital ECG database. This study was determined to be exempt from review by the institutional review board in accordance with institutional policy. Patients were required to have at least one recorded ECG in AF during a previous non‐COVID‐19 hospitalization, in addition to at least one ECG during a COVID‐19 hospitalization to be included in the study. Patients with implanted electronic pacing devices were excluded from this study, even if intermittent native atrioventricular (AV) conduction was present.

### 2.2 ECG analysis

Pre‐COVID‐19 ECGs with heart rates (HRs) that were within 10 beats per minute of the COVID‐19 ECGs were selected for comparison. Only ECGs obtained during hospitalization were included. For intubated patients, ECGs were collected prior to intubation where patients were not under the influence of sedatives or sympathomimetics. Mean HR and each RR interval in milliseconds (ms) were measured using electronic on‐screen calipers. HRV was assessed by calculating time‐domain measurements, including standard deviation of successive differences in RR intervals (SDSD), root mean square of successive differences in RR intervals (RMSSD), and the proportion of number of pairs of successive RR intervals that differ by more than 50 ms (pNN50). These measurements were calculated based on formulas previously defined and validated in the literature.^8^

### 2.3 Clinical characteristics and outcomes

The electronic health record of patients was reviewed to collect relevant baseline clinical characteristics, including age, gender, beta‐blocker, calcium channel blocker, and antiarrhythmic drug use. Laboratory data, including COVID‐19 inflammatory and cardiac injury markers, were collected. Data on hospital length of stay (LOS), need for admission to intensive care unit (ICU), ICU LOS, need for endotracheal intubation, and mortality were collected to assess and compare clinical outcomes. Clinical outcomes during COVID‐19 were correlated to HRV. This was achieved by dividing the cohort into quartiles based on SDSD, RMSSD, and pNN50 to allow for comparison. Patients were categorized as having reduced HRV if their average time‐domain indices were in the lowest quartile of patients in the cohort. Patients were classified as having preserved HRV if their average time‐domain measurements were in the upper three quartiles of patients in the cohort.

### 2.4 Statistical analysis

Each patient served as their own control. Means of continuous variables were analyzed using an independent sample t test, and categorical variables were analyzed using the chi‐square test. Kaplan–Meier curves and the log‐rank test were used to compare survival stratified by HRV. A two‐sided *P*‐value of <.05 was used to determine statistical significance. Analyses were performed using STATA/SE 16.1 (College Station, TX, USA).

## 3 RESULTS

A total of 38 patients were included in the study. There were 184 ECG tracings available for analysis, 95 ECGs from 44 pre‐COVID‐19 hospitalizations and 89 ECGs from 38 COVID‐19 hospitalizations. The pre‐COVID‐19 hospitalization diagnoses include the following: decompensated congestive heart failure (8), atrial fibrillation with rapid ventricular response (7), chronic obstructive pulmonary disease exacerbation (5), myocardial infarction (4), cerebrovascular accident (4), pneumonia (4), gastrointestinal hemorrhage (2), sepsis (3), septic arthritis (2), pyelonephritis (2), acute kidney injury (1), hypertensive urgency (1), and cholecystitis (1). Table 1 shows the baseline characteristics during the COVID‐19 admission of all included patients.

**TABLE 1:** Baseline clinical and laboratory characteristics of 38 patients

Characteristic	All patients (N = 38)
Age in years	78.60 (11.37)
Male gender	17 (44.73%)
Hypertension	29 (76.30%)
Diabetes mellitus	29 (76.30%)
Chronic obstructive pulmonary disease	12 (31.58%)
Chronic kidney disease	18 (47.37%)
Coronary artery disease	6 (15.89%)
Congestive heart failure	20 (52.63%)
Active cancer	5 (13.16%)
Beta‐blocker use during COVID‐19 admission	27 (71.05%)
Calcium channel blocker use during COVID‐19 admission	13 (34.21%)
Antiarrhythmic drug use during COVID‐19 admission	4 (10.52%)
Beta‐blocker use pre‐COVID‐19	28 (73.68%)
Calcium channel blocker use pre‐COVID‐19	11 (28.95%)
Antiarrhythmic drug use pre‐COVID‐19	4 (10.52%)
Peak CRP mg/dL	13.45 (11.89)
Peak D‐dimer ug/mL	2981.83 (7325.92)
Peak ferritin ng/mL	1121.59 (1253.89)
Peak creatine kinase IU/L	171.28 (206.83)
Peak procalcitonin ng/mL	1.97 (5.82)
Peak INR	2.02 (1.16)
Peak hs‐TnT ng/L	74.81 (90.46)
Peak pro‐BNP pg/mL	6371.04 (8555.44)
Peak fibrinogen mg/dL	546.88 (214.35)
Peak interleukin‐6 pg/mL	1731.13 (3605.50)
Peak absolute neutrophil count B/L	10.46 (7.79)
Nadir of absolute lymphocyte count B/L	0.62 (0.47)

Note

CRP C‐reactive protein, INR international normalized ratio, hs‐TnT high‐sensitivity troponin T, pro‐BNP pro‐brain natriuretic peptide. Data presented as number (%) or mean (standard deviation).

### 3.1 ECG analysis

Table 2 compares the ECG characteristics of patients during their pre‐COVID‐19 admissions versus during their COVID‐19 admission. Notably, the values for SDSD, RMSSD, and pNN50 were all smaller during COVID‐19 (*P* <.001), indicating reduced HRV. Importantly, there was no difference in the use of beta‐blockers, calcium channel blockers, or anti‐arrhythmic drugs (*P* >.05) in all patients when comparing pre‐COVID‐19 versus COVID‐19 hospitalization.

**TABLE 2:** Comparison of ECG characteristics during pre‐COVID‐19 versus COVID‐19 admissions

ECG characteristic	Pre‐COVID‐19 admission (N = 95)	During COVID‐19 admission (N = 89)	*p*‐value
Heart rate in beats per minute	95.89 (24.33)	101.57 (22.81)	0.10
Standard deviation of successive differences in ms	109.03 (50.63)	90.26 (37.18)	<0.001
Root mean square of successive differences in ms	184.06 (80.36)	147.26 (59.75)	<0.001
pNN50 in %	73.79 (16.33)	65.60 (16.60)	<0.001

Note

Data presented as mean (standard deviation).

### 3.2 Clinical characteristics and outcomes stratified by degree of heart rate variability

**FIGURE 1:**
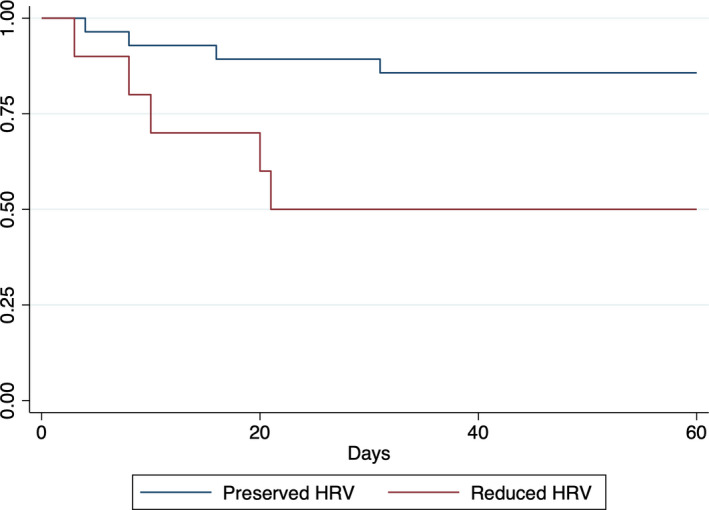
Kaplan–Meier survival curve stratified by pNN50. HRV, heart rate variability

Table 3 demonstrates the clinical outcomes of patients during COVID‐19 hospitalization stratified by degree of HRV. Patients with reduced HRV (lowest quartile) had an associated increase in mortality when stratified by pNN50 (50.00% vs. 14.29%; *P* =.02), compared with patients with preserved HRV (upper three quartiles). Of the nine patients that died, eight were due to COVID‐19 pneumonia. One patient died of unknown etiology. The Kaplan–Meier analysis in Figure 1 demonstrates that those with reduced pNN50 were less likely to have survived after a follow‐up of 60 days (log‐rank test *P* =.02). However, mortality was not associated with stratification by SDSD and RMSSD. There was no difference in the need for admission to the ICU, need for nonsurgical intubation, hospital LOS, or ICU LOS between the two groups when stratified by any index of HRV.

**TABLE 3:** Clinical outcomes stratified by degree of heart rate variability during COVID‐19 hospitalization

Outcome	Preserved heart rate variability (upper three quartiles)	Reduced heart rate variability (lowest quartile)	*p*‐value
Standard deviation of successive differences	N = 28	N = 10	
Hospital length of stay in days	12.89 (7.65‐18.14)	14.90 (6.78‐23.02)	0.68
Intensive care unit length of stay in days	5.43 (0.46‐10.39)	8.40 (0.66‐17.46)	0.53
Admitted to intensive care unit	14 (50.00%)	4 (40.00%)	0.59
Non‐surgically intubated	11 (39.29%)	4 (40.00%)	0.97
Deceased	7 (25.00%)	2 (20.00%)	0.75
RMSSD	N = 26	N = 12	
Hospital length of stay in days	12.12 (7.00‐17.23)	16.25 (7.70‐24.80)	0.36
Intensive care unit length of stay in days	5.69 (0.34‐11.05)	7.33 (0.14‐14.80)	0.72
Admitted to intensive care unit	13 (50.00%)	5 (41.67%)	0.63
Nonsurgically intubated	10 (38.46%)	5 (41.67%)	0.85
Deceased	7 (26.92%)	2 (16.67%)	0.49
pNN50	N = 28	N = 10	
Hospital length of stay in days	13.54 (8.32‐18.75)	13.10 (4.65‐21.55)	0.93
Intensive care unit length of stay in days	7.46 (1.86‐13.07)	2.70 (0.09‐5.31)	0.31
Admitted to intensive care unit	14 (50.00%)	4 (40.00%)	0.53
Nonsurgically intubated	10 (38.46%)	5 (50.00%)	0.43
Deceased	4 (14.29%)	5 (50.00%)	**0.02**

Note

Data presented as mean (95% confidence interval) or number (percentage %).

Additionally, when stratified by any of the time‐domain measures of HRV, there was no difference in age, gender, the use of AV nodal blocking medications (beta‐blockers, calcium channel blockers, or antiarrhythmic drugs) pre‐COVID‐19 or during the patients’ COVID‐19 admission, or any laboratory values (peak value of: C‐reactive protein, D‐dimer, ferritin, creatine kinase, procalcitonin, international normalized ratio, high‐sensitivity troponin T, pro‐brain‐type natriuretic peptide, fibrinogen, and absolute neutrophil count; nadir of absolute lymphocyte count).

## 4 DISCUSSION

This study demonstrates that patients with cAF have reduced HRV during COVID‐19 compared with prior illnesses. This observation was not related to differences in usage of AV nodal blocking drugs between pre‐COVID‐19 and COVID‐19 admissions. Kaplan–Meier survival analysis demonstrated an association between reduced pNN50 and mortality. However, there was no difference in mortality when patients were stratified by other time‐domain measures of HRV. There was no difference in any other clinical outcome or characteristic between patients stratified by HRV.

The heart is abundantly innervated by autonomic nerves, with autonomic control consisting of intrinsic and extrinsic ganglia.^9^ The extrinsic sympathetic control of the heart is largely mediated by the cervical, cervicothoracic, and thoracic ganglia. The extrinsic parasympathetic control of the heart is largely mediated by the vagus nerve.^10^, ^11^, ^12^ Clusters of intrinsic ganglia that are composed of ganglionated plexi are located in the atria, where they are innervated by adrenergic and vagal nerve endings close to the pulmonary vein ostia.^10^, ^11^, ^12^, ^13^ These clusters of intrinsic ganglia regulate the interactions between the extrinsic and intrinsic nervous systems.

Heart rate variability analysis is widely used as a noninvasive method to characterize the influence of the ANS on sinus rate.^14^ Increases in parasympathetic tone are believed to increase HRV, where vagal withdrawal reduces HRV.^14^, ^15^ Hence, low HRV reflects reduced cardiac parasympathetic tone and is a significant predictor of adverse cardiac outcomes in studies where autonomic modulation of the sinus node has been studied.^16^, ^17^, ^18^, ^19^ Lower HRV has been demonstrated in a number of conditions, including coronary artery disease, congestive heart failure, diabetes, and hypertension.^20^, ^21^, ^22^ In sinus rhythm, high HRV demonstrates a favorable cardiovascular adaptive response to various endogenous and exogenous factors.^23^ AF itself is marked by high HRV, which is secondary to the pattern of AV conduction in AF by influencing the conduction properties of the AV node.^24^, ^25^, ^26^ Reduced HRV in patients with AF has been shown to be an independent predictor of cardiovascular and all‐cause mortality.^27^ Multiple reports have described new‐onset AF in patients diagnosed with COVID‐19, but the electrophysiologic impact of COVID‐19 in patients with established cAF has not been described.^28^, ^29^ Patients with cAF characteristically demonstrate marked HRV, primarily reflecting the refractoriness of the AV node. The depressed HRV that we observed in cAF patients with COVID‐19 compared with their previous illnesses may be reflective of detrimental impact on the AV node due to COVID‐19. It is possible that COVID‐19 affects important components involved in the ANS control of the heart, including the GPs and AV node.

Several important mechanisms may explain how COVID‐19 can adversely affect the heart. The SARS‐CoV‐2 virus that causes COVID‐19 uses the angiotensin converting‐enzyme 2 (ACE2) to enter target cells.^30^ ACE2 is expressed in the epithelium or endothelium of multiple organs, including the heart and blood vessels. Cleavage of angiotensin I to angiotensin II by ACE promotes vasoconstriction and pro‐inflammatory as well as pro‐oxidative effects via the angiotensin II receptor type 1.^30^ ACE2 itself leads to anti‐inflammatory, anti‐oxidative, and vasodilatory effects through the angiotensin 1‐9‐Mas receptor complex. Thus, internalization of the virus causes downregulation of ACE2 on the cell surface, promoting endothelial dysfunction, vascular inflammation, and protective signaling pathways in cardiac myocytes.^30^, ^31^ Other plausible mechanisms of myocardial injury include damage to cardiac myocytes by respiratory failure and hypoxemia, coronary microvascular thrombosis due to hypercoagulability, and from host inflammatory response.^32^, ^33^ Hyperinflammation and cytokine storm can cause myocarditis through pathologic T cells and monocytes.^30^ Additionally, the presence of antiphospholipid antibodies, including anticardiolipin, has been seen in COVID‐19 patients.^34^, ^35^ It has been proposed that anticardiolipin antibodies affect the cardiac conduction system by mediating an antigen–antibody reaction.^36^ Indeed, anticardiolipin antibodies have an established pathogenic role in rheumatologic conditions such as systemic lupus erythematosus, where AV conduction failure is seen.

### 4.1 Limitations

Our study was limited by a small sample size; hence, it was difficult to reveal correlations between additional clinical outcomes between groups stratified by degree of HRV. There are inherent limitations to the retrospective nature of our study. ECG tracings collected were 10 seconds in duration as our study was conducted in a real‐world cohort of patients with active COVID‐19 infection. Thus, we were unable to collect tracings that are 5 minutes to 24 hours in duration, which is the gold standard. However, the time‐domain indices of HRV described in this study have been collected with durations of ECG tracings less than 5 minutes by multiple research groups, where recording periods in the range of seconds are reported to be acceptable.^37^

## 5 CONCLUSION

This is the first study to describe the electrophysiologic effect of COVID‐19 on AV conduction in patients with cAF. We found that in patients with cAF, HRV was reduced during COVID‐19 compared with prior illnesses. This observation occurred irrespective of the use of AV nodal blocking drugs. Importantly, patients with the most reduced HRV was associated with increased mortality when stratified by pNN50. Depressed HRV in AF may reflect changes in autonomic control of AV conduction, and if confirmed, may be another marker of cardiac injury in COVID‐19.

## CONFLICT OF INTERESTS

The authors declare that they have no competing interests. The results presented in this paper have not been published previously in whole or part, except in abstract form.

## AUTHORS’ CONTRIBUTIONS

BP design, manuscript, supervision. JJ data collection, analysis, manuscript. JR data collection, analysis, manuscript.

## ETHICS APPROVAL AND CONSENT TO PARTICIPATE

All procedures performed in studies involving human participants were in accordance with the ethical standards of the institutional and/or national research committee and with the 1964 Helsinki declaration and its later amendments or comparable ethical standards. This study was determined to be exempt from review by the institutional review board in accordance with institutional policy. This article does not contain any studies with animals performed by any of the authors.

## Data Availability

Data are safely kept in a password‐protected security system at Thomas Jefferson University Hospital. The data sets used and/or analyzed during the current study are deidentified and available from the corresponding author on reasonable request.
